# Is There Any Difference in Stem Cell Population between Type I and Type II Endometrial Cancer? A Pilot Study

**DOI:** 10.34763/jmotherandchild.20252901.d-24-00041

**Published:** 2025-05-24

**Authors:** N Muthuraman, Anitha Thomas, Thomas Samuel Ram, KM Mohankumar, Premila Abraham

**Affiliations:** Associate Professor, Department of Biochemistry, Christian Medical College, Vellore, Tamil Nadu, India; Professor, Department of Gynecologic Oncology, Christian Medical College, Vellore, Tamil Nadu, India; Professor, Department of Radiotherapy, Christian Medical College, Vellore, Tamil Nadu, India; Scientist, Centre for Stem Cell Research (CSCR), Bagayam, Vellore, Tamil Nadu, India

**Keywords:** Endometrial Neoplasms, Neoplastic Stem Cells, Tumour Biomarkers, Aldehyde Dehydrogenase, Nanog Homeobox Protein

## Abstract

**Background:**

The incidence of endometrial cancer is increasing globally. Cancer stem cells are now considered the driving force for tumour recurrence and metastasis. We studied whether the proportion of cancer stem cell population and stemness gene expression differ in type I and type II endometrial cancer.

**Materials and methods:**

Type I and type II endometrial tumour tissues were obtained from patients who underwent hysterectomy. The tumour tissue was digested using collagenase, and we established a primary culture. In the primary cultures established from these two types of cancer, we used flow cytometry to measure the proportion of the cancer stem cell population expressing CD 133 and CXCR4 on its surface. We also looked for the expression of genes related to stemness, regulators of stemness, and markers of metastasis in both these cancer types.

**Results:**

We found that the proportion of cancer stem cell population that expresses CD133 and CXCR4 was higher in type II endometrial cancer than in type I endometrial cancer. Also, genes (Nanog, ALDH, EZH2) related to stemness and aberrant transcriptome were found to be upregulated in type II endometrial cancer.

**Conclusion:**

Our study demonstrates that the proportion of stem cells in type 2 endometrial cancer is higher than in type I endometrial cancer. The findings of this study should lead us to investigate with a larger sample size and see if the increase in the stem cell population in type II endometrial cancer may be the reason for its poor prognosis.

## Introduction

Endometrial cancer is one of the most common gynaecological malignancies in the world [1]. The incidence of endometrial cancer is increasing worldwide due to the increased prevalence of risk factors such as obesity and an ageing population [2]. Increased body mass index is considered to be one of the risk factors for the development of endometrial cancer [3]. Obesity is shown to increase the oestrogen levels in the blood and can also lead to a pro-inflammatory state in the endometrium; both of these factors play a crucial role in the pathogenesis of endometrial cancer [4,5]. Based on histological characteristics, endometrial cancer is broadly divided into type I and type II endometrial cancer [6]. Grade I or grade II endometroid adenocarcinoma fall under type I endometrial cancer, which is generally associated with a good prognosis. Grade III endometroid adenocarcinoma, undifferentiated carcinoma, carcinosarcoma, serous and clear cell fall under type II endometrial cancer, whose prognosis is poor as compared to type 1 endometrial cancer [7]. Type 1 tumours are oestrogen dependent and are more common, whereas type II tumours are oestrogen independent and are less common [8].

Tumour tissue is heterogeneous in nature, encompassing varied types of cells. Cancer stem cells or tumour initiating cells are a unique population of cells in a tumour tissue which retains embryonic character [9]. Cancer stem cells are attributed as the chief cause of drug resistance, increased invasiveness and metastatic potential of tumours [10,11]. Cancer stem cells also lead to treatment relapse [12]. The origin of these cancer stem cells in a tumour remains a mystery, since a tissue resident stem cell, a terminally differentiated cell in a tissue and at times even treatment mediated senescent cells can get transformed into a cancer stem cell upon accumulating the necessary mutations such as overexpression of oncogenes and inactivation of tumour suppressor genes [13,14]. Normal stem cells interact with the niche, which is composed of cells that can send both growth signals and inhibitory signals as and when required to keep the proliferation of stem cells in check. Cancer stem cells may have mutations that enable them to multiply despite the absence of growth signals or the presence of inhibitory signals from the niche. Similarly, mutations in the cells present in the niche can also transform a resident stem cell in a tissue into cancer stem cells [15].

CD133 is a membrane glycoprotein in cancer stem cells of many solid tumours like prostate, ovary, lung, brain, colon, and type I and II endometrial cancer. CD133^+^ cells in type I endometrial cancer had increased migration, proliferating potential, stemness features and chemo-resistant property compared to CD133^−^ cells [16]. CXC motif chemokine receptor 4 (CXCR4), a stromal cell-derived factor-1 receptor, was elevated in many tumour cells. CD133^+^CXCR4^+^ markers were a promising surface marker in isolating cancer stem cell populations from endometrial cancer. These double positive populations showed increased chemoresistance as compared to other subpopulations of cells in endometrial cancer [17].

An exciting perspective about the risk of tumour development in a particular tissue was unravelled, which ascertains that the number of stem cells and their proliferation rate in a tissue is positively correlated with tumour development very strongly [18]. Considering the poor prognosis of type II endometrial cancer compared to type I endometrial cancer, we speculated that the proportion of the cancer stem cell population may be higher in type II endometrial cancer, and the expression of key genes involved in the maintenance of cancer stemness, its invasiveness, and metastatic potential can be different in type I and type II endometrial cancer tissue.

## Materials and methods

### Cell culture

HEC1B cell line, Endometrial Carcinoma; Human (Homo sapiens) was procured from American Type Culture Collection (ATCC-HTB-113). The cells were cultured in Eagle’s Minimum Essential Medium (ATCC, EMEM Catalog No. 30-2003) supplemented with 1x Pen-Strep (Gibco™) and 10% fetal bovine serum FBS (Gibco™). Media was changed every 3 days and upon reaching 90% confluence, cells were subcultured using 0.25% trypsin.

### Endometrial tumour tissue digestion and establishment of primary culture

The Institutional Review Board approved this study. Endometrial tumour samples were obtained from patients who gave informed consent to participate in the study. Tumour tissue was obtained from type I and type II endometrial carcinoma patients who underwent hysterectomy, the tumour tissue was divided in such a way that a portion of it goes to pathology lab for examination and the remaining portion was transported to the tissue culture lab in Dulbecco’s Modified Eagle Medium (DMEM, Gibco) supplemented with 1x Pen-Strep. Tumour tissue was washed with PBS to remove blood and debris. Tumour tissue was minced using a sterile scalpel blade into tiny fragments of 1 mm^3^. Enzymatic digestion mixture containing collagenase type IV (Sigma-Aldrich, C5138) 30 mg and hyaluronidase (Sigma-Aldrich, H1115000) 600U in 10 mL of DMEM was used to incubate the minced tumour tissue at 37°C for 30 min. Undigested and large tumour tissues were filtered using a 40 μm sterile mesh filter. The filtrate containing cells was washed twice with culture media by centrifugation at 200 g for 10 min at 25°C. Cell number and viability were assessed using trypan blue dye staining in the hemocytometer. Tumour cells at a density of 50,000 cells/ml in culture media were plated in a 100 mm Petri dish.

### Quantification of gene expression in a patient sample

Total RNA from the primary cells established from the tumour sample were isolated using the NucleoSpin RNA kit (Machari-Nagel) and reverse transcribed using the cDNA Synthesis Kit (iScript™ Bio-Rad). The relative expression (ΔΔCT) of *Oct, Sox, Nanog, KLF4, cMYC, EZH2, ALDH, TNNT, BMI1 and S100A4* genes was determined using the respective primers **(Table 1)** with quantitative real-time PCR (qRT-PCR) (Applied Biosystem Quant Studio Flex 6).

**Table 1.** List of primers used and their sequence

**Genes**	**Primer sequence**
GAPDH-F	5′-CTGACTTCAACAGCGACACC-3′
GAPDH-R	5′-TAGCCAAATTCGTTGTCATACC-3
β Actin-F	5′-CCAGAGCAAGAGAGGCATCC-3′
β Actin-R	5′-CCGTGGTGGTGAAGCTGTAG-3′
SOX2-F	5′-CCATCCACACTCACGCAAAA-3′
SOX2-R	5′-TATACAAGGTCCATTCCCCCG-3′
OCT4-F	5′-TCCCATGCATTCAAACTGAGG-3′
OCT4-R	5′-CCAAAAACCCTGGCACAAACT-3′
NANOG-F	5′-TGGACACTGGCTGAATCCTTC-3′
NANOG-R	5′-CGTTGATTAGGCTCCAACCAT-3′
BMI 1-F	5′-AATCCCCACCTGATGTGTGT-3′
BMI 1-R	5′-GCTGGTCTCCAGGTAACGAA-3′
c-MYC-F	5′-AAACACAAACTTGAACAGCTAC-3′
c-MYC-R	5′-ATTTGAGGCAGTTTACATTATGG-3′
KLF 4-F	5′-ATCTTTCTCCACGTTCGCGTCTG-3′
KLF 4-R	5′-AAGCACTGGGGGAAGTCGCTTC-3′
EZH2-F	5′-CCCTGACCTCTGTCTTACTTGTGGA-3′
EZH2-R	5′-ACGTCAGATGGTGCCAGCAATA-3′
ALDH1A1-F	5′-CGGGAAAAGCAATCTGAAGAGG-3′
ALDH1A1-R	5′-CCCGCTCAACACTCCTTCGA-3′
S100A4-F	5′-CCACAAGTACTCGGGCAAAG-3′
S100A4-R	5′-GTCCCTGTTGCTGTCCAAGT-3′
TNNT1-F	5′-GGTCAAGGCAGAACAGAAGC-3′
TNNT1-R	5′-GCGGTTGTACAGCACGTTGA-3′

**Table 2:** Patient details

**Hospital No**	**Age**	**Biopsy findings**	**Endometrial cancer type**
639043B	56	Endometrioid adenocarcinoma Grade I-II	Type 1 endometrial cancer
707263H	60	High grade endometrioid adenocarcinoma (poorly differentiated) Grade III	Type 2 endometrial cancer

### Quantification of tumour spheres

HEC 1B cells were sorted into CD133^+^ and CD133^−^ cells using CD133 antibody (Miltenyi biotec, CD133 MicroBead Kit – Tumour Tissue, human Cat No: 130-100-857) in a magnetic activated cell sorter as per instructions laid out in the kit inlet. For tumour sphere formation, HEC 1B cells were grown in Eagle’s Minimum Essential Medium, (ATCC, EMEM Catalog No. 30-2003) supplemented with 1x Pen-Strep (Gibco™), Human Recombinant epidermal growth factors (20 ng/mL, EGF, STEMCELL Technologies 78006.1), Human Recombinant Basic fibroblast growth factor (20 ng/mL. bFGF, STEMCELL Technologies 78003_C) in the absence of fetal bovine serum. Cells were plated in 96 well plate at a seeding density of 3000 cells/well. Each group (CD133^+^, CD133^−^ and unsorted) of cells were plated as triplicates. Spheres measuring more than 50μM were counted using microscopy on day 7.

### Flow cytometry

Primary endometrial cancer cells derived from a patient tumour sample were stained with APC Mouse Anti-Human CD133 (BD Biosciences, 566596) and PerCP-Cy™5.5 Mouse Anti-Human CD184 (BD Biosciences, 560670) for 30 min at 4°C. The proportion of CD133^+^ and CXCR4^+^ cells was analysed using BD FACS Aria III flow cytometer.

## Statistics

The statistical tests were performed using GraphPad Prism 8.1. The normality of the data was tested using the Shapiro-Wilk test. One-way ANOVA was followed by post hoc Bonferroni correction to identify the significant difference between individual groups. In all the tests, p<0.05 was considered statistically significant.

## Results

### Stem cell population in the HEC1B cell line

**Figure 1.**
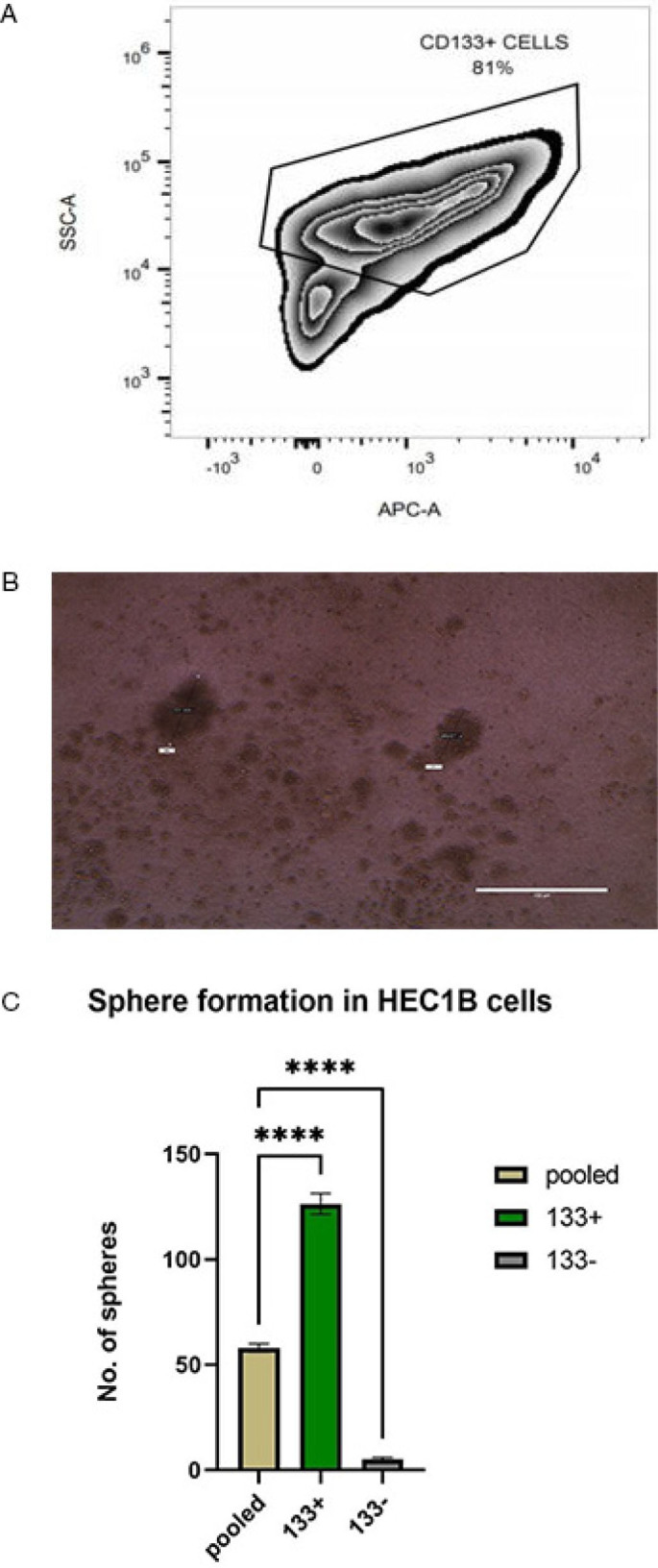
Represents the stemness markers and sphere-forming ability of HEC1B. (A) Flow cytometric analysis of CD133^+^ cells in the HEC1B cell line. (B) Spheres formation in HEC1B cells grown in serum-free media. (C) Sphere forming ability of pooled, CD133^+^ and CD133^−^ cells grown in serum-free media. ****p<0.001.

Our aim was to identify the stem cell population in the HEC1B (Human endometrial cancer) cell line. CD133-expressing cells were considered to have stemness features in endometrial cancer. We used flow cytometry to identify the CD133^+^ cells in HEC1B (Fig. 1A). The cells were then sorted into CD133^+^ and CD133^−^, using a magnetic activated cell sorter. Sphere formation is one of the key properties of cancer stem cells. Three subsets of cells were cultured, which includes pooled, CD133^+^ and CD133^−^ cells in the appropriate media and looked for their sphere forming ability (Fig. 1B). As expected, the sphere forming ability of CD133^+^ cells were much higher as compared to CD133^−^ and pooled cells (p<0.001) (Fig. 1C). We can infer that stem cells are present in HEC1B cell line and CD133^+^ cells can be considered as stem cell population, due to its higher sphere forming ability.

### Proportion of stem cell population in type I and type II endometrial cancer

**Figure 2.**
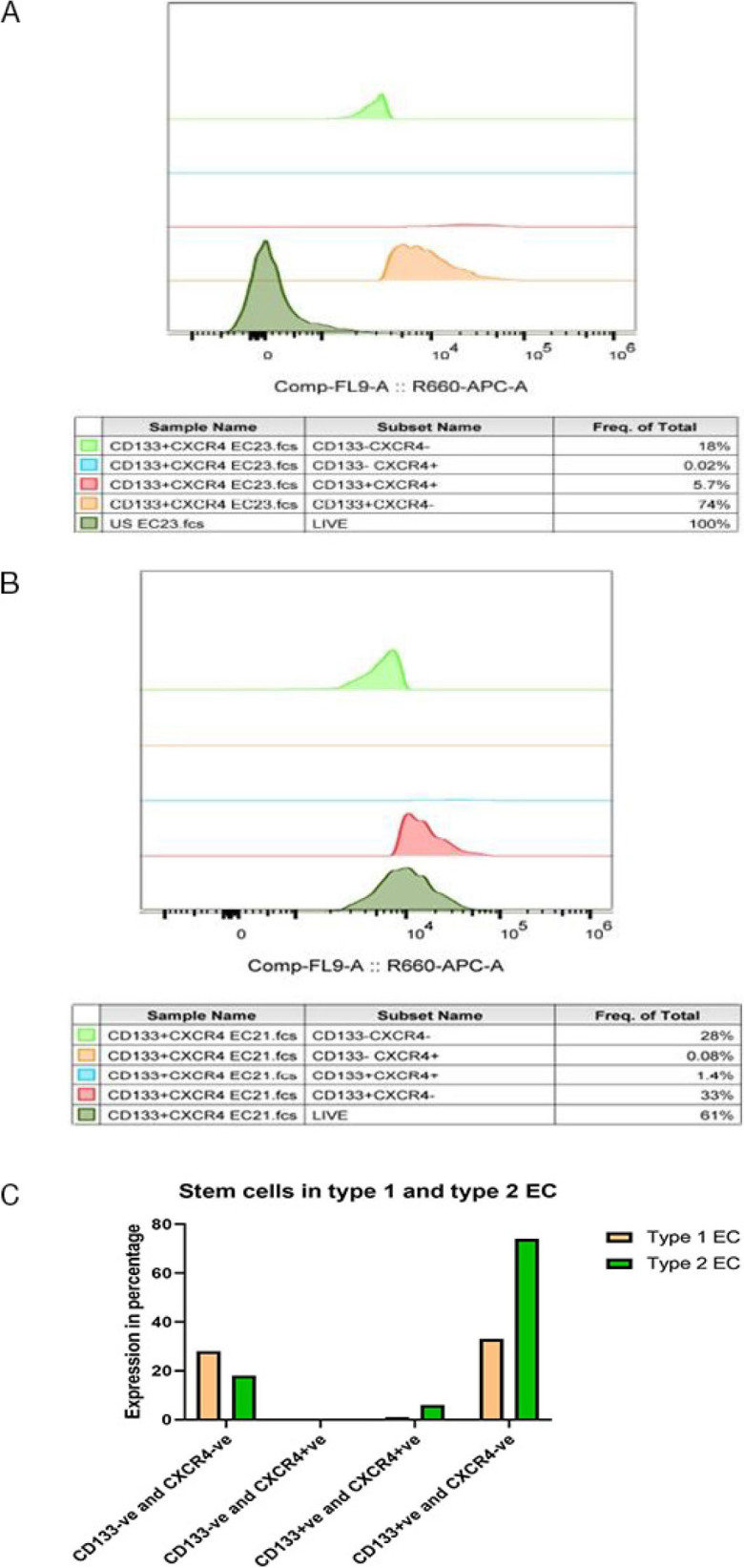
Represents the percentage of different fractions of cells as analysed by flow cytometry. (A) Proportion of different fractions of cells in type I endometrial cancer. (B) Proportion of different fractions of cells in type II endometrial cancer. (C) Flow cytometric analysis of the percentage of different fractions of cells in type I and type II endometrial cancer tumour samples.

Having ascertained the stemness property of CD133^+^ cells, we were interested in seeing the proportion of cells expressing this marker in type I and type II endometrial cancer. Since CD133^+^CXCR4^+^ endometrial cancer cells were shown to possess more significant proliferation, clonogenic, tumourigenic, and chemoresistance abilities (17), we also analysed the proportion of these double positive cells in primary cell culture established from patient’s tumour sample diagnosed to be type I endometrial cancer and in other patient’s tumour sample diagnosed to be type 2 endometrial cancer (Fig. 2A & 2B). The proportion of suspected cancer stem cell population (CD133^+^CXCR4^+^ and CD133^+^CXCR4^−^) was found to be higher in primary cells established from type II endometrial tumour sample (5.7% and 74%) as compared to primary cells established from type I endometrial tumour sample (1.4% and 33%) **(Fig. 2C)**. This signifies that the cancer stem cell population is higher in type II endometrial cancer as compared to the less harmful type I endometrial cancer.

### Markers of stemness in type I and type II endometrial cancer

**Figure 3.**
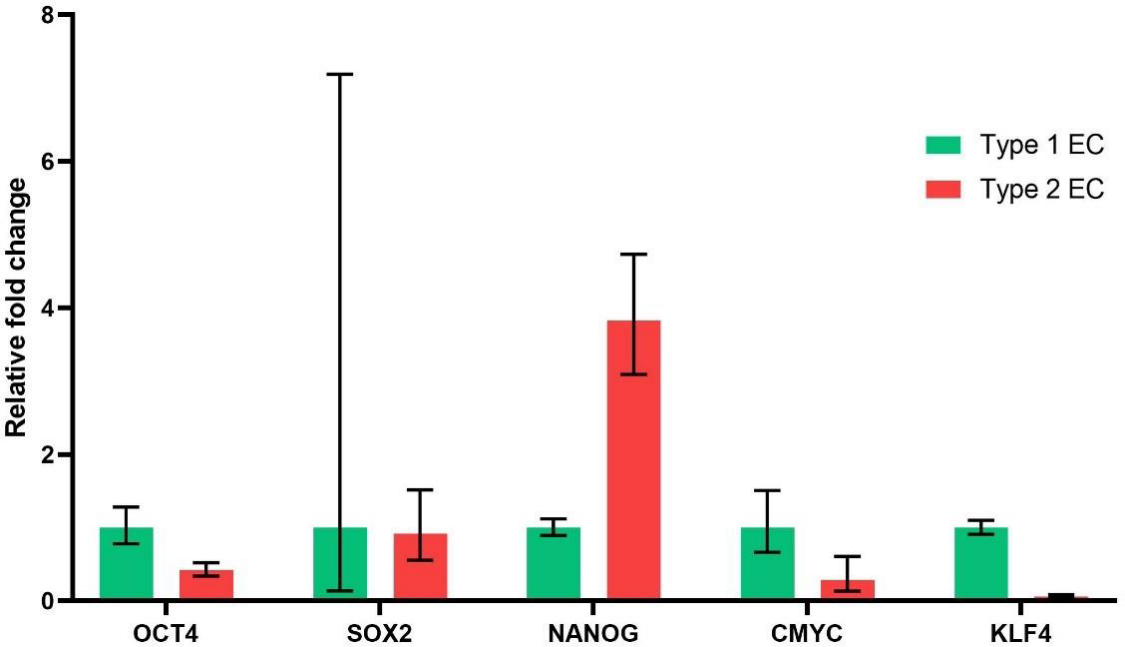
Expression of genes related to stemness in type 1 and type 2 endometrial cancer. Expression of genes related to stemness in type 1 and type 2 endometrial cancer.

Expression of transcription factors OCT4, SOX2, Nanog, KLF4 and cMYC is considered a marker of stemness. These stemness markers were abnormally expressed in different types of tumours. In addition, higher expression of these stemness markers was found to be associated with poor survival outcomes in many tumours [19]. High expression of Nanog was shown to be a stimulus for acquiring stemness and epithelial to mesenchymal transition, which is a crucial developmental process in cancer cells to obtain invasive characteristics [20]. So, we analysed the difference in expression of these stemness genes in type I and type II endometrial cancer cells obtained from patients. We observed higher expression of the Nanog gene in type II endometrial cancer cells as compared to type I endometrial cancer cells (Fig. 3). The expression of other genes related to stemness, such as OCT4 and SOX2, was found to be similar in both types of endometrial cancer. The expression of cMYC and KLF4 was found to be upregulated in type I endometrial cancer as compared to type II endometrial cancer.

### Genes involved in the regulation of cancer stemness in type I and type II endometrial cancer

**Figure 4.**
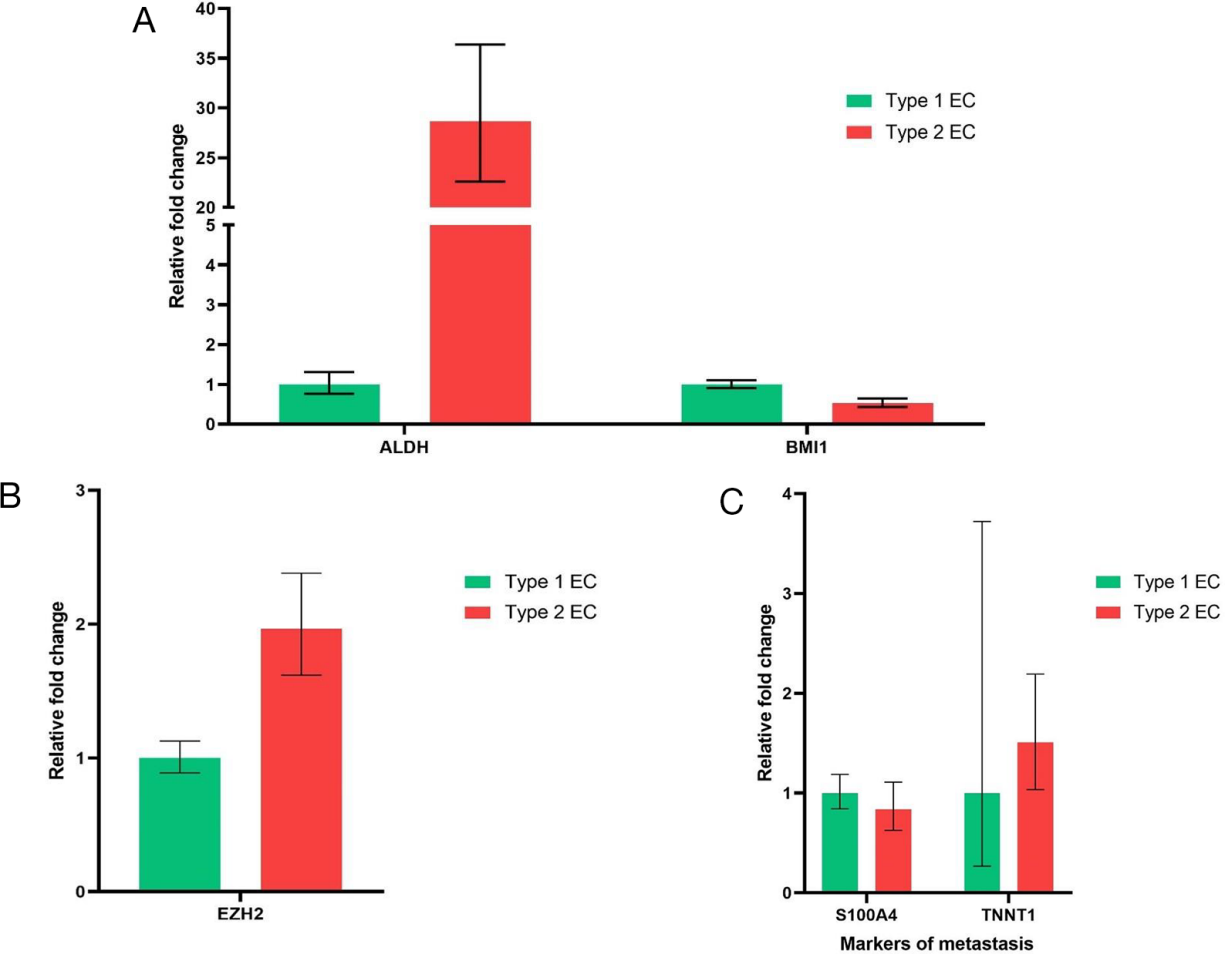
Expression genes related to maintenance of stemness and metastasis. (A) Expression of ALDH1A1 and Bmi1 in type 1 and type 2 endometrial cancer. (B) Expression of EZH2 in type 1 and type 2 endometrial cancer. (C) Expression of markers of metastasis in type 1 and type 2 endometrial cancer.

Next, we checked whether genes involved in the maintenance of stemness in a particular tissue are differentially expressed in type I and type II endometrial cancer. We considered two important candidate genes to assess this. Aldehyde dehydrogenase 1A1 (ALDH1A1) and B-cell-specific Moloney murine leukemia virus insertion site 1 (Bmi1), which is a member of the Polycomb group (PcG) gene family. ALDH1A1 is involved in the maintenance of stemness in both normal and cancer stem cells. ALDH1A1 upregulation was associated with increased tumour aggressiveness, treatment resistance, and poor prognosis in many tumours [21]. BMi1 regulates chromatin structure by silencing crucial genes and maintains stemness in cancer cells [22]. Increased expression of Bmi1 was shown to be associated with poor prognosis in endometrial cancer [23]. Bmi1 expression was found to be similar in both types of endometrial cancer (Fig. 4A). Expression of ALDH1A1 was found to be upregulated several folds higher in type II endometrial cancer as compared to type I endometrial cancer (Fig. 4A). This can be inferred that ALDH1A1, the key gene involved in maintenance of stemness is upregulated in type II endometrial cancer and could be the reason for increased percentage of stem cells seen in type II endometrial cancer as compared to type I endometrial cancer.

### Gene involved in initiating aberrant transcription and metastasis in cancer

We looked for the expression of genes notorious for promoting aberrant transcription in type I and type II endometrial cancer. Enhancer of zeste homolog 2 (EZH2) is a member of polycomb group genes (PcGs), which facilitates epigenetic modifications to increase the transcription of oncogenes and suppress the transcription of tumour suppressor genes. Increased expression of EZH2 was shown in many solid cancers and was associated with poor prognosis [24]. Endometrial cancer cells obtained from type II endometrial cancer showed upregulated expression of EZH2 as compared to type I endometrial cancer ( Fig 4B). We also checked for the expression of genes involved in facilitating metastasis in endometrial cancer, such as S100A4 and TNNT1. S100 A4 belongs to the family of calcium-binding proteins S100, whose expression was a marker of invasion and metastasis in prostate and endometrial cancer (25). Troponin T1 (TNNT1) is one of the subunits of troponin T (TNT), which is required for contraction and relaxation of muscle. TNNT1 expression was upregulated in some of the invasive forms of colorectal and breast cancer (26). However, the expression of these metastatic genes, S100A4 and TNNT1, was similar in both type I and type II endometrial cancer. (Fig. 4C).

## Discussion

Tumour cells are heterogeneous in nature. The cancer stem cell hypothesis explains that there is a hierarchy in which the self-renewable cancer stem cells can give rise to various progenitor cells, and that in turn can lead to proliferation of varied terminally differentiated cells [27]. Cancer stem cells are resistant to chemotherapy by acquiring numerous mutations which favour the expression of multidrug-resistant ABC transporter proteins, tilting the balance towards antiapoptotic signals to evade apoptosis [28]. A terminally differentiated cell, upon acquiring mutations, can dedifferentiate into a cancer stem cell [29]. Cancer stem cells can also transdifferentiate, in which there can be a shift from one lineage of cell to another to favour a suitable niche for the cancer cells to grow [30]. In our study, we isolated cancer stem population from HEC1B using CD133 as a marker and demonstrated its stemness nature using its sphere-forming ability, since tumour sphere formation is considered one of the unique properties of cancer stem cells [31].

It was clearly shown using a machine learning approach (one-class logistic regression machine learning algorithm - OCLR) that increased stemness phenotype can lead to all adverse outcomes like metastasis and recurrence in varied tumour tissues [32]. Our study is in concordance with this finding, showing a higher proportion of cancer stem cell population (CD133^+^ and CD133^+^, CXCR4^+^) in type 2 endometrial cancer with poor prognosis as compared to type 1 endometrial cancer with good prognosis. Cancer stem cells are shown to alter the niche in a way that favours tumour progression even in unfavourable conditions and also enhances the metastatic and invasion potential [33].

The presence of cancer stem cells in the tumour tissue is further substantiated by increased expression of embryonic-specific transcription factors such as OCT4, SOX2, Nanog, cMYC and KLF4 in tumour tissues [34]. We found that the expression of the embryonic transcription factor Nanog was increased severalfold in type 2 endometrial cancer compared to type 1 endometrial cancer. Nanog expression was also reported to be elevated in many solid tumours, such as liver cancer, breast cancer, and colorectal cancer. [35]. Overexpression of Nanog was shown to facilitate the process of epithelial-mesenchymal transition (EMT), which can lead to the invasive nature of the tumour cells. The expression of other embryonic-specific transcription factors was not upregulated in our type 2 endometrial cancer patient.

Aldehyde dehydrogenase (ALDH) enzyme is expressed in higher levels in cancer cells and cancer stem cells resistant to chemotherapy. This enzyme, being a detoxifying enzyme, can prevent the damage caused by chemotherapy to cancer stem cells [36]. As expected, the primary cells cultured from type 2 endometrial cancer patients showed much higher expression of ALDH1A1 than type 1 endometrial cancer cells. Higher expression of ALDH1A1 probably favours the survival of cancer stem cells in type 2 endometrial cancer. Also, increased expression of the ALDH1 gene is associated with poor survival outcome in endometrial cancer [37]. The epigenetic regulator polycomb group gene EZH2, which causes aberrant transcription and enhances stemness [38] in tumour tissue, was upregulated in type II endometrial cancer as compared to type I endometrial cancer. However, the markers of invasion S100A4 and TNNT1 were found to be similar in both types of cancer at this stage of disease.

### Study limitations

The limitation of this study is that the findings are based on observations from just one patient each in type I and type II endometrial cancer patients; this must be checked in a suitable sample size to generalise these findings for the diseased population. However, this study helps us in appreciating the fact that the stem cell population is higher in type II endometrial cancer, and further studies should be done to substantiate these findings and see whether this could be the cause for the relapse and poor prognosis. Future studies are required to see if analysing these stem cell populations in each patient can help us tailor the therapy to prevent recurrence and improve survival in endometrial cancer patients.

### Key points

➢ The proportion of cancer stem cell population is higher in type II endometrial cancer as compared to type I endometrial cancer.
➢ Markers of embryonic stemness, the Nanog gene and the master regulator of stemness in solid malignancy, ALDH1A1 gene expression, were higher in type II endometrial cancer than type I endometrial cancer.
➢ EZH2, the gene which causes aberrant transcription and stemness, was also found to increase expression in type II endometrial cancer.

## List of abbreviations

CXCR4: CXC motif chemokine receptor 4
ALDH1A1: Aldehyde dehydrogenase 1A1
OCT4: Octamer-binding transcription factor 4
SOX2: SRY (sex determining region Y)-box 2
KLF4: Kruppel-like factor 4
EZH2: Enhancer of zeste homolog 2
S100A4: S100 Calcium Binding Protein A4
TNNT1: Troponin T1
